# Poverty dynamics, poverty thresholds and mortality: An age-stage Markovian model

**DOI:** 10.1371/journal.pone.0195734

**Published:** 2018-05-16

**Authors:** Shayna Fae Bernstein, David Rehkopf, Shripad Tuljapurkar, Carol C. Horvitz

**Affiliations:** 1 Department of Biology, Institute for Theoretical and Mathematical Ecology (ITME), University of Miami, Coral Gables, FL, United States of America; 2 School of Medicine, Division of Primary Care and Population Health, Stanford University, Stanford, CA, United States of America; 3 Department of Biology, Stanford University, Stanford, CA, United States of America; University of California Irvine, UNITED STATES

## Abstract

Recent studies have examined the risk of poverty throughout the life course, but few have considered how transitioning in and out of poverty shape the dynamic heterogeneity and mortality disparities of a cohort at each age. Here we use state-by-age modeling to capture individual heterogeneity in crossing one of three different poverty thresholds (defined as 1×, 2× or 3× the “official” poverty threshold) at each age. We examine age-specific state structure, the remaining life expectancy, its variance, and cohort simulations for those above and below each threshold. Survival and transitioning probabilities are statistically estimated by regression analyses of data from the Health and Retirement Survey RAND data-set, and the National Longitudinal Survey of Youth. Using the results of these regression analyses, we parameterize discrete state, discrete age matrix models. We found that individuals above all three thresholds have higher annual survival than those in poverty, especially for mid-ages to about age 80. The advantage is greatest when we classify individuals based on 1× the “official” poverty threshold. The greatest discrepancy in average remaining life expectancy and its variance between those above and in poverty occurs at mid-ages for all three thresholds. And fewer individuals are in poverty between ages 40-60 for all three thresholds. Our findings are consistent with results based on other data sets, but also suggest that dynamic heterogeneity in poverty and the transience of the poverty state is associated with income-related mortality disparities (less transience, especially of those above poverty, more disparities). This paper applies the approach of age-by-stage matrix models to human demography and individual poverty dynamics. In so doing we extend the literature on individual poverty dynamics across the life course.

## Introduction

In 2014, 14.8% of the U.S. population lived below the poverty threshold [1]. In that year, the official poverty threshold for a family of four was an annual income of $24,008. If a family’s annual income falls below a threshold, all the individuals in the family are considered below the threshold as well. It is now widely accepted that those in poverty have higher mortality risk then those above poverty [2]. Yet how many of these people stay below the official poverty threshold the next year? As the ‘official’ poverty threshold is set very low, negative effects of relatively low income are also seen for individuals “near” poverty, variously defined as 1.25, 1.5, and 2× ‘official’ poverty, all the way up to the median income level, which is approximately 3× the ‘official’ poverty threshold. For this reason we define 3 possible “poverty” thresholds, 1×, 2×, and 3× the ‘official’ poverty threshold set by the U.S. Census Bureau as the poverty level. We compare annual survival, remaining life expectancy, and entry and exit of individuals above and below each poverty threshold at each age. We also investigate how three cohorts, each with one of the three specified poverty thresholds experience dynamic heterogeneity, that is, how the demographic structure of the population varies as individuals cross in and out of poverty at each age.

It is well documented that income levels are dynamic, thus being “in poverty” is also dynamic [3]. Of those classified as poor in 2009, for example, 26.9% were classified as not being poor in 2010 and 35.4% were classified as not being poor in 2011. Of those not poor in 2009, 4.1% were poor in 2010 and 5.4% did become poor in 2011 [4]. Other income levels are dynamic as well, such as 2×poverty threshold ($48,016 for a family of four in 2014) and 3×poverty threshold ($72,024 for a family of four) and have important health [5], and policy ramifications as well [6].

Our objective is to answer the following four questions: 1) How often in their life course do individuals cross above and below each threshold? 2) How does being above or below a threshold affect the probability of survival from one age to the next? 3) How does being above and below a particular poverty threshold change the expected fate of a cohort, such as the remaining life expectancy and variance in remaining life expectancy? 4) How many total years are spent above and below each threshold during an individual’s life?

These questions are examined by using a stage-by-age matrix model to analyze empirical data from the National Longitudinal Survey of Youth 1979 (NLSY79) and the Health and Retirement Survey (HRS)[7, 8]. In this model demographic fates of individuals depend upon both stage (income status) and age. From NLSY79 and HRS we are able to estimate one-year survival probabilities and one-year transition probabilities across three different poverty thresholds, for ages 22-95. Our results are consistent with current literature on poverty entry and exit rates (such as [1, 4, 6]) and near poverty entry and exit rates [9].

Our method of using stage-by-age models (with two states, above poverty and in poverty for each of the three defined poverty thresholds) leads us directly to the variance in average remaining life expectancy at each age and the heterogeneity in income state. Matrix age-by-stage models have been used in many contexts (for instance to analyze plants, such as a perennial shrub [10], animals, for instance whales [11], humans [12], and in epidimiological analysis, for instance to analyze rubella [13]) and are useful for connecting individual stochasticity in life path to overall cohort dynamics. Other methods that use the combination of age and stage to predict demographic fates, include multistate life table approaches (as reviewed by Willekens [14] or classic increment-decrement life tables techniques [15]) can address similar questions. In deference to work on multi-state life table analysis, we use the human demography convention and use the term ‘state’ rather than the more general ecology term ‘stage’. Our approach serves as a bridge between age-by-stage matrix models and poverty dynamics over the life-course.

### Dynamic heterogeneity in income level and mortality risk

The association of poverty with poor health is well-documented, including different theories of mechanisms and pathways of causation [6, 16–18]. Cellini [6] also summarizes different modeling approaches for poverty dynamics (for example the tabulation or count method, life table method, bivariate hazard rate method, multivariate hazard rate (or spell based) method, components-of variance method, and some less used multivariate methods). Regardless of technique used, the association between mortality risk and poverty status is apparent, but what about an association at higher income levels? Rehkopf [19] looked at different income levels and their associated mortality risk for individuals in the United States between the ages of 18-77 and found that the greatest mortality risk is for the “population whose family income is below the median (equal to $20,190 in 1991, 3.2 times the poverty level)”. In other words mortality risk decreased as income increased until near the median income level, above this level their was no significant change in mortality risk with income increase. Thus there are income related disparities in mortality risk up to the median income.

The “near poverty” threshold, usually defined officially as 1.25× the poverty threshold (although sometimes defined as 1.3×, 1.5×, or 2× the poverty threshold) has also received attention [5, 9]. Individuals with incomes just above the poverty threshold have characteristics quite similar to those “in poverty” in terms of assistance program participation rates. Furthermore transitions into and out of “near poverty” are frequent [9]. Most studies have not looked at the full range of age-specific annual survival and transition rates, despite general trends across the life cycle. For instance, we know “[Health] Disparities are smallest during childhood, adolescence, and early adulthood and greatest in middle age, becoming weaker again in older populations” [20]. In order to better address health disparities it is useful to know the age-specific dynamics and associated mortality risk of being below different thresholds, and here we separately investigate 1×, 2×, and 3× the “official” poverty threshold. A better understanding of individual income dynamics enables a clearer identification of those most at risk.

### Age-by-stage matrix model

We construct three matrix models, one for each of the three poverty thresholds (1×, 2× and 3× the “official” poverty threshold); each matrix is a discrete state, discrete age, Markov chain matrix with two-income states, above or below the chosen threshold, at each age. The matrix is similar in structure to Tuljapurkar’s [21] population projection age-stage matrix but here, as Steiner had done [22], there is no reproduction. It is also similar in concept, to the model of multi-state mathematical demography first presented in Rogers [23]. Matrix methods have since become more widespread as the data necessary to construct them have become more available and insights they can provide are expanding [24]. Here we use our age-by-stage models to trace dynamic heterogeneity in a cohort, that is, individual state switching (above and below poverty) and cohort heterogeneity (variance in state structure) at each age (also termed individual stochasticity since individual life course trajectories are stochastic and differ even between identical individuals [11]). As is conventional with matrix methods [10], we also use the age-by-stage matrices to construct fundamental matrices from Markov Chain theory to determine remaining life expectancy for individuals in a given state at a given age and to determine expected remaining years in each income state (for theory see S1 Theory).

We address the following research questions:

For each of the three threshold income levels (1×, 2×, and 3× the “official” poverty level) we ask:

- How does annual survival probability change with age for those above and below a particular poverty threshold? What are the age-specific entry and exit probabilities for the two states (above and below a particular threshold)?
- How does state-structure (proportion above and below the threshold) change with age?
- How does remaining life expectancy and expected remaining life below the specified income threshold change with age? What is the variance in remaining life expectancy for those below and above the income threshold?
- How does the poverty status and survival of simulated individuals change across their lifetimes, where the age-specific probabilities of survival and state transitions of the model are used to assign fates to individuals in a simulated birth cohort. Specifically we ask, at what ages do individuals transition below and above a particular threshold? And how long do simulated individuals spend in poverty during their lifetime?

Our state-by-age model answers our research questions by emphasizing age and state structured cohort dynamics for the three different thresholds. We examine how age-specific individual stochasticity affects overall cohort dynamics.

## Methods: Theory

The age-by-state matrix, **L**, calculates cohort dynamics and individual trajectories. Specifically, **L** is a square matrix whose dimension equals the number of states times the number of age classes (note: a maximum age must be set). It is used: (1) to project a vector **n** that represents the number of individuals in each state at each age from birth across the lifetime [25], which enables tracking of the state-distribution and survivorship of an initial cohort (with its initial state distribution, **n(0)**) at each age *x*, as described by the following equation:

$$\begin{array}{c} n\left(x\right)=L^{x}n\left(0\right) \end{array}$$ (1)

where the matrix **L** is raised to the *x*th power at each age *x*. (2) to analyze remaining life expectancy (mean and variance in age at death) and generate individual stochastic trajectories across all ages, where an individual is a realization or sample path of a Markov process. The Markov chain is described by this matrix [26]:

$$P=\left(\frac{L}{m}|\frac{0}{1}\right)$$ (2)

Here we will define the age-and-stage matrix **L**, and in the appendix (S1 Theory) we show how it can be used in (1) and (2). Each element in **L** is composed of the product of two of the following four functions at each age, *s*_1_(*x*),*s*_2_(*x*),*t*_12_(*x*) and *t*_22_(*x*):

- *s*_1_(*x*) = Probability that an individual whose income is below a threshold survives between ages *x* and *x* + 1 (one-period state-specific survival).
- *s*_2_(*x*) = Probability that an individual whose income is above a threshold survives between ages *x* and *x* + 1 (one-period state-specific survival).
- *t*_21_(*x*) = Conditional probability of exiting poverty before age *x* + 1 for an individual who is in poverty at age *x*. (1 − *t*_21_(*x*)) = *t*_11_(*x*) Probability that an individual whose income is in poverty (below the income threshold) at age *x* will remain in poverty at age *x* + 1, conditional on survival.
- *t*_22_(*x*) = Conditional probability of staying above poverty at age *x* + 1 for an individual who is above poverty (the particular threshold) at age *x*. (1 − *t*_22_(*x*)) = *t*_12_(*x*) Probability that an individual whose income is above poverty at age *x* will enter poverty by age *x* + 1, conditional on survival.

Here *x* is a 1-year age interval (so age *x* to *x* + 1 is a one year step). Although other interval lengths can be used [27]. State transition probabilities (transitioning to a new state or staying in a state at the next age) are conditional on survival; ‘state’ is being below or above a particular income threshold. Subscripts follow standard convention of row, *i*, and then columns, *j*, and transitions are *j* to *i* (i.e. *t*_12_ is transitioning from state 2 to 1, richer to poorer). Also note that every probability has a complement; the complement to the annual state specific survival is the annual state specific mortality, i.e. the probability of dying in one year.

Multiplying the survival probabilities by the conditional state transition probabilities results in a matrix for each age, **Q(x)** which takes the following form (Table 1):

**Table 1:** Structure of state transition matrices, Q(x).

	State at age x		
State at age x+1		Below income threshold	Above income threshold
	Below income threshold	*s*_1_(*x*)*t*_11_(*x*)	*s*_2_(*x*)*t*_12_(*x*)
	Above income threshold	*s*_1_(*x*)*t*_21_(*x*)	*s*_2_(*x*)*t*_22_(*x*)

We denote each (unconditional) state transition matrix as **Q(x)**, and each state transition matrix is inserted into the sub-diagonal of the age matrix at the appropriate column. In our implementation of the model, we set a maximum age of 100 and thus there are 100 state transition matrices, each representing a 1-year increment from 0 to 100 years of age. The age-state block matrix has dimensions 101 × 101 blocks, each block is comprised of a 2 × 2 matrix, and has the following form (where the 0’s represent 2 × 2 matrices comprised entirely of zeros):

$$\begin{array}{c} L=[\begin{array}{cccccccc} 0 & 0 & 0 & \ldots & 0 & 0 & 0 & 0\\ Q(1) & 0 & 0 & \ldots & 0 & 0 & 0 & 0\\ 0 & Q(2) & 0 & \ldots & 0 & 0 & 0 & 0\\ 0 & 0 & Q(3) & \ldots & 0 & 0 & 0 & 0\\ \vdots & \vdots & \vdots & \ddots & \vdots & \vdots & \vdots & \vdots \\ 0 & 0 & 0 & \ldots & Q(98) & 0 & 0 & 0\\ 0 & 0 & 0 & \ldots & 0 & Q(99) & 0 & 0\\ 0 & 0 & 0 & \ldots & 0 & 0 & Q(100) & 0 \end{array}] \end{array}$$ (3)

The age-state block matrix **L** has a structure reminiscent of a Leslie matrix from which fecundity has been removed, in that an individual always transitions to the next age at each time step [26, 28, 29]. Since there are 2 state classes and 101 age classes, the dimensions of the age-state matrix **L** is 202 × 202. The **L** matrix can be used to produce cohort projections (see section A of S1 Theory) and markov chain analysis. With markov chain analysis we calculate the average remaining life expectancy, average remaining life in poverty (below an income threshold), and the variance (by using the fundamental matrix, see section B of S1 Theory) and the simulated individual trajectories (see section C of S1 Theory).

## Methods: Empirical data

### HRS RAND

The Health and Retirement Study (HRS) is a publicly accessible longitudinal household survey data set for the study of retirement and health among individuals over age 50 and their spouses in the United States. We use the RAND HRS Data files Version O which “are a cleaned, processed, and streamlined collection of variables derived from HRS” [8]. The survey consists of 6 cohorts and we use longitudinal data compiled from 6 of the 11 interview waves that fall approximately around these years: 2002, 2004, 2006, 2008, 2010, 2012. As a nationally representative data set of 37, 319 individuals, HRS has over-sampled Hispanics, Blacks, and residents of Florida, and provides weighting variables to make it representative of the community-based (non-institutionalized) population. For our purposes we subset the data to only include individuals between 50 and 95 years old. We use survey sample weight in all analyses. And the income threshold that defines poverty is from the United States Census Bureau (thresholds vary by year, size of family, and number of children). Individuals are pooled into repeated observations for each of their interview responses as explained after the following section.

### NLSY1979

The NLSY79 Cohort is a longitudinal project that follows the lives of a sample of American youth born between 1957-64. The cohort originally included 12,686 respondents ages 14-22 when first interviewed in 1979; after two sub-samples were dropped, 9,964 respondents remain in the eligible samples [7]. We use data available from interview wave 1 (1979 survey year) to interview wave 25 (2012 survey year), this includes one year intervals from 1979-1994, and two-year intervals from 1994-2012. Since we are studying the state of poverty we subset the data to include observations from age 22 (when individuals enter the work-force post college) to age 50. Retention rates for NLSY79 respondents from 1979 to 1993 exceeds 90 percent. Rates from 1994 until 2000 exceeded 80 percent. Rates from 2002 until 2012 have been in the 70s. (Retention rate is calculated by dividing the number of respondents interviewed by the number of respondents remaining eligible for interview) [7]. More detailed information about retention rates can be found at NLSY79’s website. Poverty rates are based on annual poverty income guidelines by the U.S. Department of Health and Family Services (which are also based on family size). We recognize that the threshold varies minimally between the U.S. Department of Health and Family Services and the U.S. Census Bureau, but previous research comparing slight differences in poverty thresholds shows the effect to be insignificant [6].

### Quantifying the functional dependence of survival and transition probabilities on age: Logistic regression

There has been much discussion as to how to calculate transition probabilities for Markov transition models [30–34]. (The latter two sources give a good background on the history of estimating transition probabilities from data and propose methods for higher Markov models). One very practical proposal for calculations of binary Markov transition models has been to use logistic regression probabilities [35]. Since logistic regression is very straightforward and intuitive, especially when we have a time-dependent covariate (age), we employ it for our analysis. Researchers might look towards [36] or [37] which are two distinct ways to calculate Markov transition probabilities that can incorporate a range of data complexities. Willekens’ review [14] is also a good resource as to the vast array of statistical packages in R that can be used to estimate transition probabilities for demographic multi-state models. Logistic regression models are often used in the poverty context to find determinants of poverty [38]. With logistic regression covariates can also be incorporated into the state-by-age model, although here we only include age and current state as our independent variables.

The NLSY79 pre-1994, NLSY post-1994, and HRS are analyzed separately for ages 22-33, 34-50, and 51-95, respectively, and the results are later concatenated to give probabilities of survival and transitioning over our complete age range of 22–95. Within each data-set interview waves are pooled together except for the last wave, this represents the data at time *t*. All the interview waves except for the first one are then pooled together to represent the data at time *t* + *i*. (To find *i* for each data-set we average the interval in months between each successive interview wave. For instance, for HRS the interval between interview years is normally distributed about 24 months, for NLSY79 pre-1994 the interval is normally distributed about 12 months, and for NLSY post-1994 there is a normal distribution in interval length about 24 months. After the regression we adjust our probabilities to be over a one year period, i.e. *t* to *t* + 1, see appendix for more on this adjustment). Instead of tracking individuals longitudinally over several ages, we perform a pooled logistic regression analysis on all observations of all ages from time *t* to *t* + *i*, where individuals have a particular age *x* at time *t* and age *x* + *i* at time *t* + *i*. If an individual did not respond at that specific point (either the interview at *t* or *t* + 1 or both interviews), that observation is omitted from the analysis. However if that specific individual responded later in the study (a different observation), that observation is included in the analysis. This technique has been called the pooled repeated observation method (PRO) and the analysis a pooled logistic regression [39]. The pooled logistic regression analysis was performed in R and the survey package [40] with svyglm was used to incorporate weights. For HRS we have 186,585 observations between the ages of 50 and 95. For NLSY79 after pooling we have 183,238 observations between the ages of 22 and 50. For analysis the pooled observations are weighted based on each data sets weight at observation.

For each income threshold and data-set we ran regression analysis to obtain estimates for the functional dependence of survival and transition probabilities on age and state. The regression coefficients (S1 Tables, Table A and B are coefficients from HRS data, Tables C, D, E and F are coefficients from NLSY79 data) were logit transformed into probability values. Since the actual time between interview waves *i* varied (see S4 Fig) and we needed to calculate annual rates from the probabilities, we developed a protocol for converting the probabilities to annual (12 month) rates (as explained in S1 Appendix). We then evaluated these functions at each age for use in our matrix models.

To check for an association of income level and response rate at each age, the difference in percent of non-response was calculated for each age. Three income level thresholds (1×, 2× and 3× the income level that “officially” defines “poverty”) were used to define binary states (above and below each threshold) and separate analyses were run for each. For this analysis deceased individuals were removed from the population (they were not counted as non-response, see S2 Appendix).

In our main analysis we investigated three income thresholds to classify individuals into “states” in order to see if the impact of income level on mortality depended upon where the line between rich and poor was drawn. Also we were interested in whether the dynamics of changing state were similar if the line was drawn at a very low income level versus a higher income level.

## Results

### How do the probabilities of survival and transitioning between income states depend upon age and current state?

We answered the first research question by performing pooled logistic regression for individuals classified into two states at each age; below and above 1×, 2×, and 3× the “official” poverty threshold. (See S2 Appendix for more on the empirical data including the distribution of non-response based on poverty state and age.)

**Fig 1:**
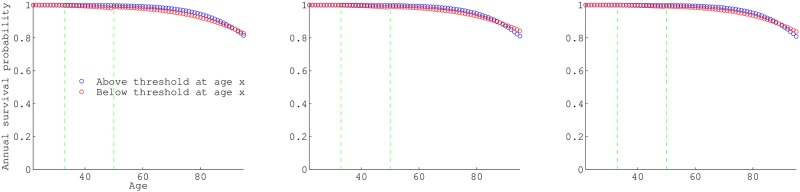
Age-specific annual survival probabilities. Annual survival (the number alive at age *x* + 1/ the number alive at *x*) for those above (red) and below (blue) the poverty threshold at age *x*. a, b and c differ in threshold income used to define poverty, 1×, 2×, and 3× the ‘official’ poverty income level, respectively. The green vertical lines in each panel are the seams between data-sets utilized: NLSY79 pre-1994, NLSY79 post-1994 and HRS (at age 33 and 50).

For ages 50 and above there is a significant difference between survival of those above and below the 1×, 2× and 3× the “official” poverty threshold (Fig 1). The richer survived better than the poorer group no matter where the threshold was set. The maximum observed difference in annual survival between the two groups (at any age) was 2.3% for the 1×poverty threshold, and 1.9% for both the 2× and 3× poverty threshold.

In terms of absolute difference, those above 1× poverty have their maximum annual survival advantage between ages 75-79 of 0.022. Those above 2× poverty have the maximum advantage also in the late 70s with a maximum difference of 0.018, and those above 3× poverty have maximum advantage at age 76 of 0.018. For the 1× poverty threshold there is a crossover at age 91 where those below threshold have a slight survival advantage. For the 2× and 3× poverty threshold the crossover is at age 88. At younger ages (22-50 from NLSY79) there is less disparity in survival probability between states (except at ages 45-50 for the 1× poverty threshold).

**Fig 2:**
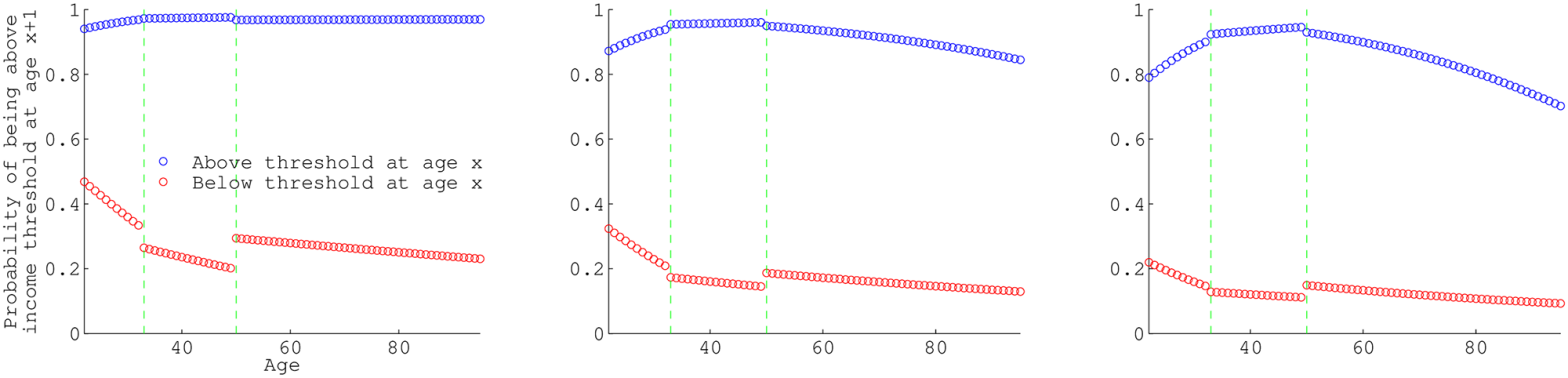
Age-specific conditional probabilities of being above the poverty threshold. Annual probability of being above the poverty threshold at age *x* + 1 for those below (red) or above (blue) the poverty threshold at age *x*, conditional on survival. a, b, and c, differ in the threshold income used to define poverty, 1×, 2×, and 3× the ‘official’ poverty income level, respectively. The green vertical lines in each panel are the seams between data-sets utilized: NLSY79 pre-1994, NLSY79 post-1994 and HRS (at age 33 and 50).

Individuals above 1× poverty income tend to stay there throughout their life course. Those below 1× poverty income have the greatest probability of exiting their low income state at younger ages. (Fig 2). Those below 2× and 3× poverty income threshold also have decreasing probability of exiting their income state, and the annual probability declines with age. Those above 2× poverty have a slight increasing probability of remaining above 2× poverty until mid-ages, where the probability of remaining above 2× poverty declines with age. Those above 3× poverty have a more dramatic increase in the probability of remaining above 3× poverty until midages as well, and then a very sharp decrease of the probability of remaining above 3× poverty with each age.

Discontinuity in the curve (at the dashed vertical lines) is a seam at the joining of NLSY79 (ages 22-50 between 1979-2012) to HRS (ages 50-95 between 2002-2012). It perhaps represents a period effect since the two data sets include observations from different time periods as well as different ages. The age-structure of interviews also differ between the data-sets, as can be seen in S3 Fig; NLSY79 is a classic cohort through time, whereas HRS has a new cohort entering the data-set in 2004 and 2010. Regardless, our adjustments to the probabilities so that they represent annual rates and the data-set’s large sample size create a general age-specific trend consistent with previous literature.

### Cohort distributions

We want to know the state structure (the relative proportion of richer and poorer individuals) at each age for any initial cohort. We note that each **Q(x)** represents the joint demographic processes of survival to the next age and transitions among income levels during that age interval. For example **Q(1)** takes any initial cohort from age 0 to age 1 and **Q(2)** takes the cohort from age 1 to age 2. The cumulative demographic processes experienced by a cohort from age 0 through age 2 is given by the product of the two, the matrix product **Q(2)Q(1)**. We note that matrix multiplication is written from right to left and that matrix multiplication is not commutative. Thus the cumulative demographic processes experienced by a cohort from age 0 to age *x* is given by the matrix product:

$$\begin{array}{c} \text{Qcum}(x)=Q(x)Q(x-1)Q(x-2)...Q(3)Q(2)Q(1) \end{array}$$ (4)

**Fig 3:**
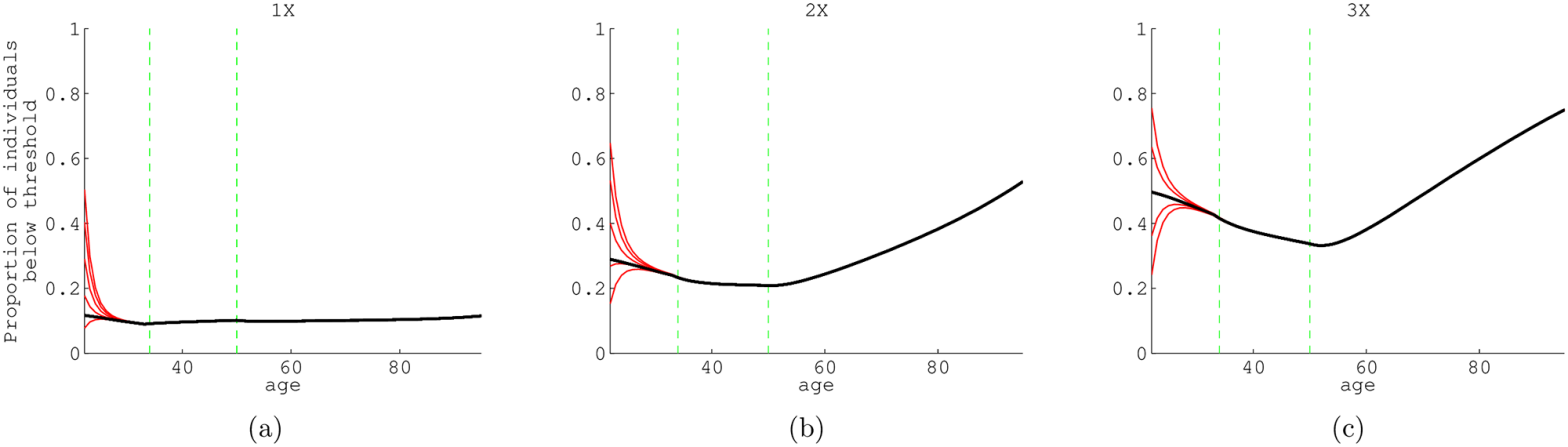
Predicted proportion of individuals that are below the poverty threshold. Black lines are the predicted proportion of individuals of age *x* that are below the poverty threshold according to the dominant eigenvector of the cumulative **Q**(*x*) matrix **Qcum**(*x*), the cumulative unconditional state transition matrix for age *x*. Red lines represent population projections for those below poverty with five different initial cohort distributions: (from top to bottom) 970,000, 750,000, 500,000, 250,000, and 30,000 individuals below poverty threshold in an initial cohort of 1,000,000 individuals. Regardless of initial cohort, the proportion of individuals below poverty (red lines) converge to the dominant eigenvector of **Qcum**(*x*) with age. a, b, and c, differ in the threshold income used to define poverty, 1×, 2×, and 3× the ‘official’ poverty income level, respectively. The green vertical lines in each panel are the seams between data-sets utilized: NLSY79 pre-1994, NLSY79 post-1994 and HRS (at age 33 and 50).

We observed that the state structure at each age appears to converge to the dominant eigenvector of this cumulative matrix product for any initial cohort, after a certain age (see Fig 3). We are not sure of the generality of this observed result. As expected, and despite underlying state switching, throughout all ages there is an almost constant percentage of individuals below 1× poverty (about 12%, Fig 3). The percentage below 2× and 3× poverty is slightly U-shaped with increasing percentages in the lower income states after age 55. (This correlates with the increased probability of exiting the higher income state after mid-ages). The demographic structure defined by the dominant eigenvectors of **Qcum(x)**, the cumulative age-specific unconditional state transition matrix, is similar to the demographic structure seen in the empirical data (S1 Fig).

We cannot refer to this as a ‘stable state’ distribution since as the cohort is being projected across time it is also changing age, and the relative number of individuals above and below the threshold changes at each age. However if the cohort would be theoretically stuck at an age for a long period of time, the dominant eigenvector of the cumulative matrices (**Qcum(x)**) represents the stable state distribution that would be approached. More precisely, if we picked an age ‘*z*’ and assumed that all **Q(x)** = **Q(z)**, then the quasi-stable [41] distribution would be the dominant eigenvector of *Q*(*z*). The subdominant eigenvalue of each cumulative unconditional transition matrix (with survival not included) represents how quickly a cohort converges to the quasi-stable distribution, and we find that convergence happens very quickly for all three thresholds. In other words, even if the initial state distribution varies, we observed that a cohort will quickly (by age 30) converge into the quasi-stable distribution represented by the dominant eigenvector of **cumQ(x)**, the cumulative stage matrices.

### The fundamental matrix: Life expectancy and variances

**Fig 4:**
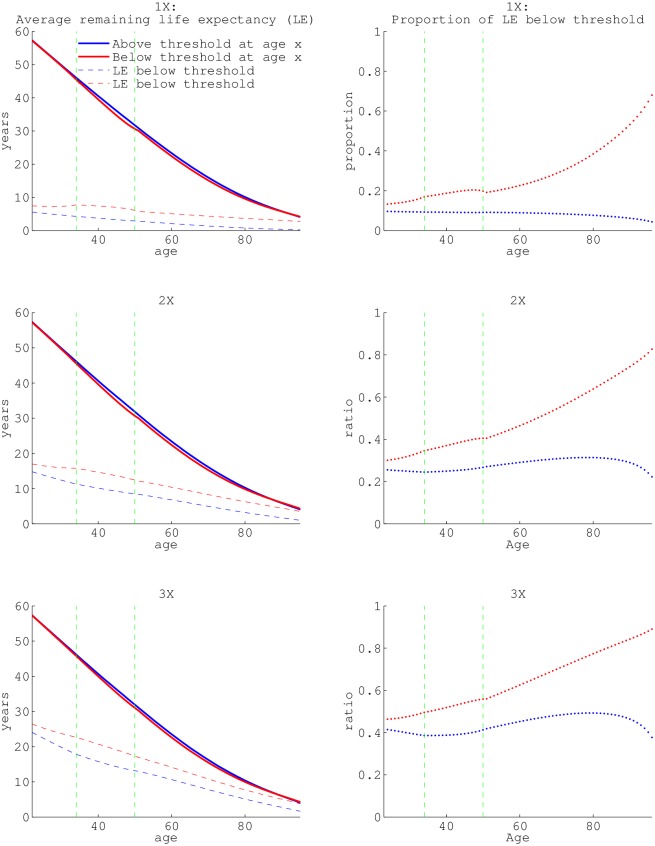
The average remaining life expectancies and remaining years in poverty. Average remaining life expectancies (solid lines) and expected years in poverty (dashed lines) conditional on surviving to age *x* based on state at age *x* (red = below threshold, blue = above threshold). a, c, and e, differ in the threshold income used to define poverty, 1×, 2×, and 3× the ‘official’ poverty income level, respectively. The ratio of the average remaining life in poverty (or below 2× poverty or 3× the ‘official’ poverty threshold) to total average remaining life are graphed in panels (b), (d) and (f). In other words, this is the proportion of average remaining life below a specified threshold. All values are calculated from the fundamental matrix (see section B of S1 Theory). The dashed green vertical lines in each panel are the seams between data-sets utilized: NLSY79 pre-1994, NLSY79 post-1994 and HRS (at age 33 and 50).

We want to know how many years individuals of a given age, in a given income level, are expected to remain alive. We also want to know how many of their remaining years will be spent in poverty. We therefore utilize the “fundamental matrix”, which is a matrix that is essentially the summation of the powers of **L** across all ages. Each element of the fundamental matrix is the mean number of visits to either poverty or above poverty (depending on the index) conditional on survival to a particular age and state [42]. Analysis of the fundamental matrix provides age-by-state-specific average remaining life expectancy, and expected remaining years below an income threshold (Fig 4). Those above ‘poverty’ at age *x* have less expected remaining years in ‘poverty’ than those already in ‘poverty’ at age *x*, for every defined threshold (Fig 4 dashed lines). As age increases, for the higher than 1× poverty income state, the average proportion of life that can be expected to be in the below 1× poverty state decreases (blue dotted lines). This is generally true for those above 2× poverty as well, however from about ages 60 to 80 the expected proportion of life below 2× poverty increases slightly (and increases much more for those above 3× poverty). Regardless of initial stage, there are fewer expected years of remaining life for those below a 1× poverty threshold than below a 2× or 3× poverty threshold.

The difference in average remaining life expectancy between the income states is greatest (for all thresholds) at mid-ages. The peak difference is 1.44 years, 1.35 years, and 1.01 years for those above and below 1×, 2× and 3× poverty, respectively. Those above and below a 1× poverty threshold have an average life expectancy difference of over 1/2 year from ages 34-81, for those above and below 2× poverty this occurs between ages 34-78, and for 3× poverty ages 37-77.

**Fig 5:**
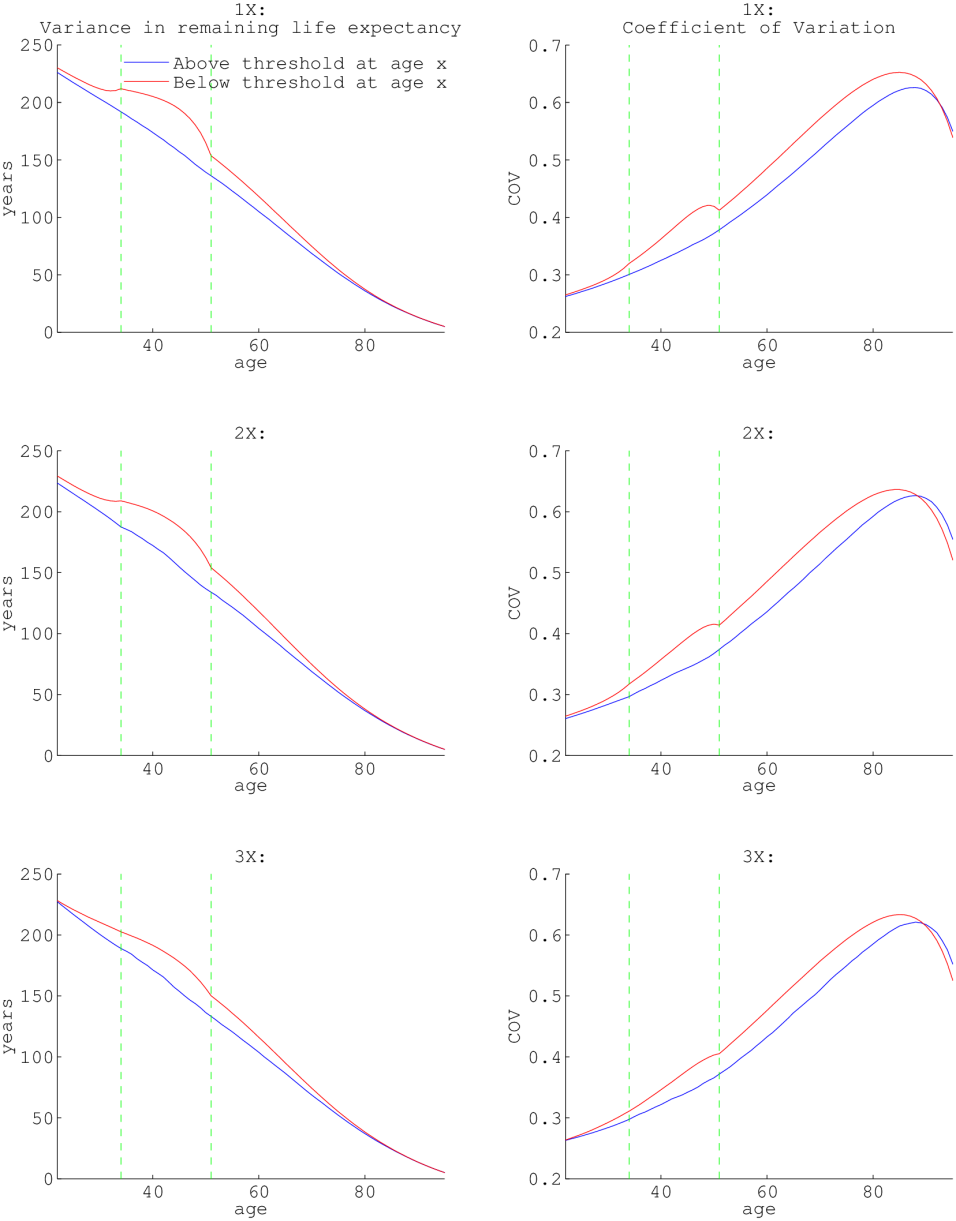
The variance of remaining life expectancy, conditional on surviving to a specific age. a, c, and e depict the variance of remaining life expectancy conditional on surviving to age *x* based on state at age *x* (red = below threshold, blue = above threshold). The age-specific coefficient of variation (the standard deviation divided by the mean) for state (red = below threshold, blue = above threshold) at age *x* are graphed in panels (b), (d) and (f). The dashed green vertical lines in each panels are the seams between data-sets utilized: NLSY79 pre-1994, NLSY79 post-1994 and HRS (at age 33 and 50).

Analysis of the fundamental matrix also facilitates quantifying the variance and coefficient of variance in average remaining life expectancy at each age. Variance in a population represents the range of possibilities individuals can experience in terms of lifespan, and in terms of time in income states. We can see that for all ages (except few in late old age), those in the lesser income state have the greatest variance in their average remaining life expectancy (Fig 5). The difference in variance is most pronounced in the mid-40s. There is a maximum difference in variance of 37.14 years for those above and below 1× poverty, 33.19 years for the 2× poverty states, and 24.42 years for the 3× poverty states. The difference between the variance for both income states is above 15 years for those between the ages of 33-57, 33-58, and 37-55 for 1×, 2× and 3× poverty thresholds, respectively. Variance scales with the mean in general. To look at variability independent of the mean, we also examined the coefficient of variation. We found that the coefficient of variation increases with age, especially more so for those in the lower income state.

### Simulation: Individual lifetime trajectories

**Fig 6:**
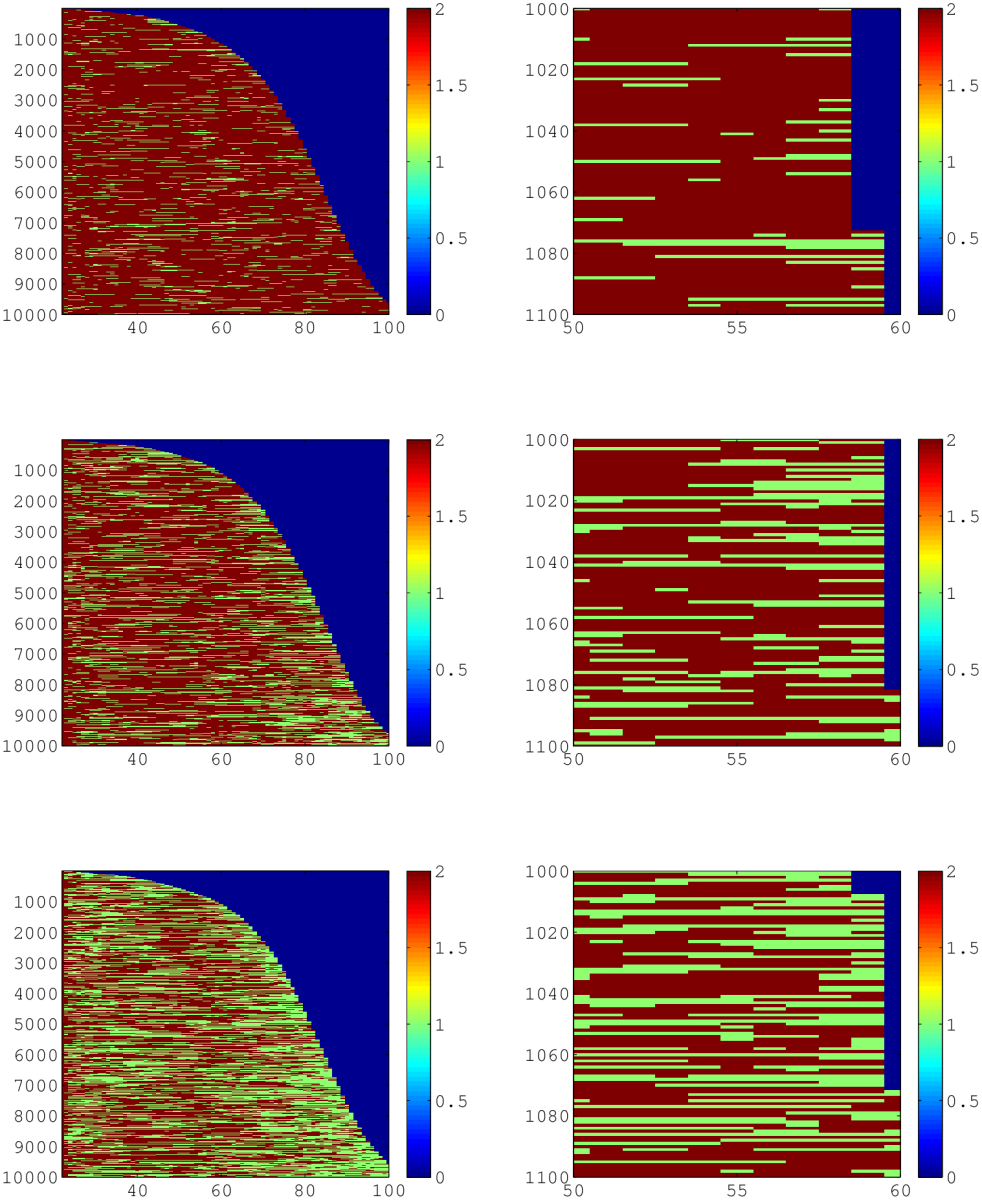
Cohort simulations of 10,000 individuals lifetime trajectories. The right column is a ‘snapshot’ of 100 individuals between the ages of 50 and 60 with mortality around 60. Red is the above income threshold state, green is below the income threshold, and blue is death. The first column is the entire cohort and is in the shape of a survivorship curve for all three rows. The first row of panels has a threshold at 1× poverty income threshold, second row is 2× and third row is 3× poverty income threshold.

**Fig 7:**
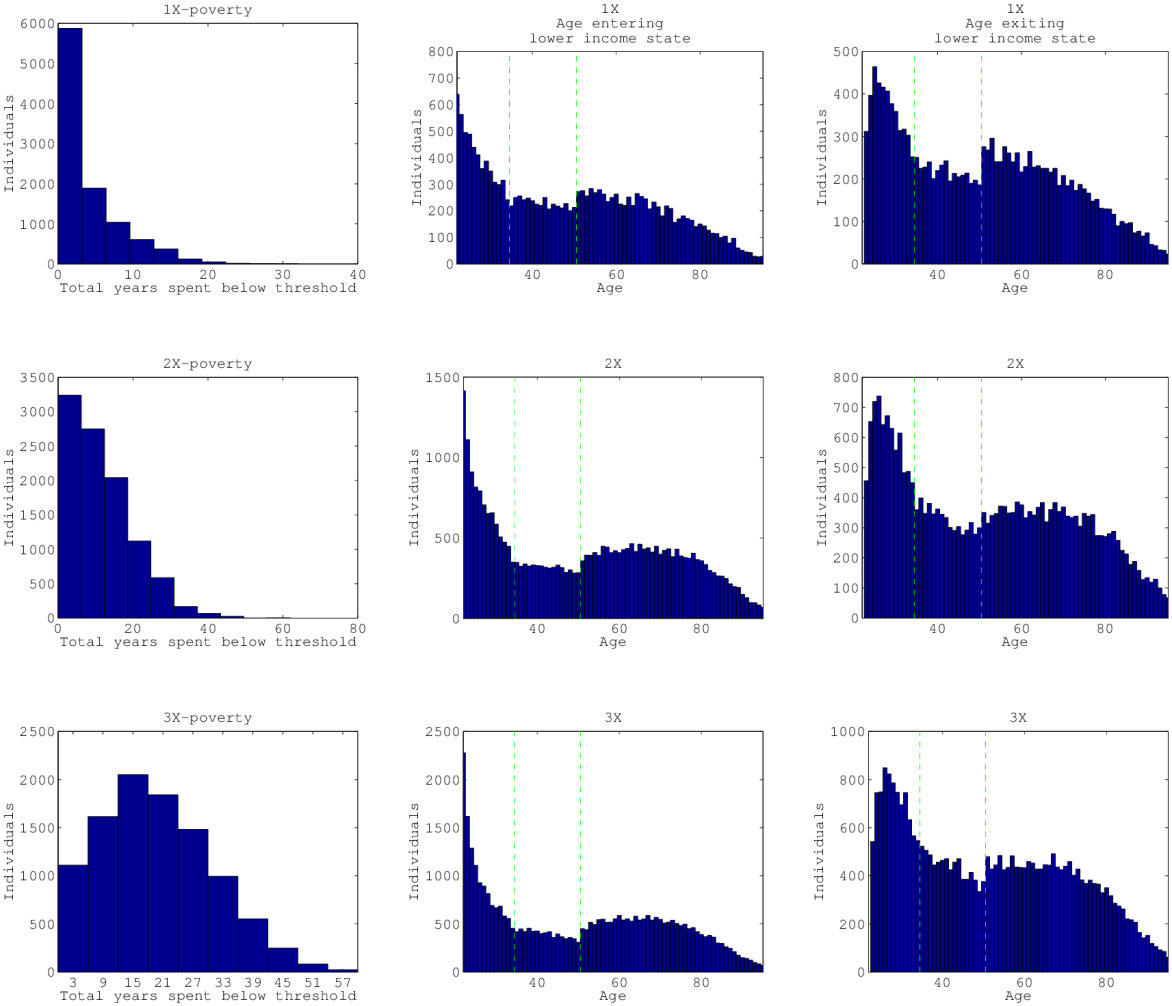
Cohort simulations of 10,000 individuals: Distributions of residence times and ages of transitions. The initial cohort is the same for each row but each row is a separate simulation with a particular income threshold in place. Distributions of the years an individual spends in the lower income state are depicted in panels a, d, and g. The panels in the second column are distributions of ages of entry into the lower income state. The panels in the third column are distributions of ages of exit from the lower income state. The first row of panels has a threshold at 1× poverty income threshold, second row is 2× and third row is 3× poverty income threshold. The dashed green vertical lines in panels in the last two columns are the seams between data-sets utilized: NLSY79 pre-1994, NLSY79 post-1994 and HRS (at age 33 and 50).

For our simulation of a cohort of 10,000 individuals, (Fig 6), we can clearly observe the age patterning and dynamic heterogeneity over the life course. We see an abundance of individuals in the below income threshold state at the end of their life, and more individuals in the above income state between the ages of 40 and 60 for all three thresholds. Most individuals enter the lower income states at early ages (Fig 7), and there is a slight peak again at age 60 for those below 2× and 3× the poverty threshold. Individuals also exit the lower income state at early ages, and the amount of individuals exiting levels out until age 70. Note that “exiting” as it is counted here can be the result of death as well.

Individuals spent a mean of 3.92 years (standard deviation of 4.50 years, mode 0 years) below 1× poverty (Fig 7). A mean of 11.64 years was spent below 2× the poverty threshold (standard deviation of 8.70, mode of 0 years). And a mean of 20.42 years was spent below 3× the poverty threshold (standard deviation of 11.4841, mode of 16 years).

### Limitations

Our model is in a discrete age, binary discrete state, discrete time Markov chain framework. The probability of remaining alive and of transitioning between richer and poorer states by the next age is dependent only upon the current age and current income level of an individual. The past history of an individual is not considered in the model, even though mortality rates and poverty transition rates have been found to be related not only to current state but also to past history in other studies [3]. Half of those who end poverty spells return to poverty in the next four years [43]. There is a significant relation between current state and future state and it is this relationship that our analysis is based on. One can consider this a theory of poverty entry and exit that has no duration dependence, a ‘neutral theory’ where whether individuals enter and exit poverty at each age depends only upon their current state of income and not on any intrinsic traits. Poverty risk is equally spread throughout a cohort such that at a given age for a given income level the same probability rules apply to all individuals. Our simulations give insight into the heterogeneity among individual life trajectories that results.

## Discussion

The fundamental causes of poverty and the pathways through which higher mortality results is under ongoing investigation [44–48]. Moreover, the relationship between income and mortality is complicated by the transience of the income state. Benzeval (2001) was able to control for initial health status and found that there is indeed a causal relationship between low income and poor health [17]. In the same study they distinguished between persistent poverty and occasional episodes. All studies that have done likewise found that long term poverty or ‘near poverty’ is a greater indicator for poor health than ‘episodic’ poverty [4, 9]. Neilson (2008) found that in Chile, at some point between 1996-2001, 30 percent of the population had income under the poverty line [49]. Under closer analysis only 9.2 percent were under the poverty line for that entire period, the remainder had experienced transient/episodic poverty. They further discovered that when the poverty line is increased, chronic poverty increases systematically while transient poverty levels out (in their case at 2× the Chilean poverty threshold). Backlund [50] and Rehkopf [19] also found that as income increases past a threshold, the health benefits associated with increased income diminishes, perhaps at the median income level.

All of these observations suggest that the transience of the poverty state has important ramifications for health outcomes, mortality risk and population dynamics. And indeed, the transience of poverty has received much attention, especially due to policy implications [4, 9, 17, 18, 51].

Here, our state-by-age model captures individual heterogeneity in entering and exiting either 1×, 2× or 3× the official poverty threshold at each age. Being above and below 1× the poverty threshold has important ramification for families, such as which government sponsored programs can be utilized, although ‘near poverty’, defined as up to 2× the poverty threshold, is recognized as a state with health consequences as well. 3× poverty is roughly similar to the U.S. median income, above which their are minimal income-related disparities in health. Our approach adds to prior work in that we observe the transience of these income thresholds at each age across the life course, and then consider the resulting disparities in one-period survival, average remaining life expectancy, and variance of remaining life expectancy.

We find that the higher income state has the highest annual survival probability from mid-ages to about age 80, with a crossover in late old age. The advantage is greatest between those above and below 1× poverty when compared to those above and below 2× or 3× poverty (1). However the age patterning for each threshold is quite similar. Those above 1× poverty have a constant high probability of staying above 1× poverty at all ages. Those above 2× poverty have the highest probability of staying above threshold at midages, and those above 3× poverty have a similiar pattern but with a steeper decline after mid-ages. For those in the lower income states, the annual probability of exiting those states declines with age. Those below 1× poverty have the highest probability of exiting at each age, but the sharpest decline with age (2). Although Willekens [14] did not investigate age-specific transition rates, the annual rates are roughly similar in magnitude for exiting poverty and above poverty, and for exiting 2× poverty and above 2× poverty. Our transition rates are also consistent with poverty entry and exit rates mentioned in [4] (although our rates are age-specific). Our poverty entry rates are also consistent with Rank [52] who used the Panel Study of Income Dynamics (PSID) and found that “individuals within the sample face a significant risk of poverty at some point during their adult lives, particularly during the early (20-40) and later (60-80) stages of adulthood”. This is what we found in our simulations, across the life-course, more individuals are in the higher income state between the ages of 40 and 60 (6). In another study using the PSID, Rank (2014) [53] also found, like our simulations, that from age 60 to 90, entry into income poverty (and asset poverty) decreases (they discuss policy implications).

Our results are also in approximate agreement with Cellini’s review of the dynamics of poverty in the U.S., “that those experiencing poverty had a roughly 1 in 3 chance of leaving poverty in any given year”[6]. In that review they also discuss some of the demographics underlying poverty exit and entry rates such as race, household size, sex of household head, and education.

Our cohort state distributions at each age shows that the number of individuals in poverty, (below the 1× poverty threshold) is almost constant with age, perhaps since an almost equal number of people exit and enter 1× poverty after age 33 (as shown in our simulations 7). The 2× and 3× poverty threshold projected cohorts have similar state-distributions at each age; a decrease in individuals in the lower income state until mid-ages and an increase thereafter, steeper for the cohort with the 3× poverty threshold (3).

We found that the greatest difference in average remaining life expectancy based on state at age *x* occurs near ages 40-60 (in agreement with [20]), regardless of which threshold is considered. Although the magnitude of the difference is greater for those above and below the 1× poverty threshold, the age pattern in life-expectancy is similar for the other thresholds as well (4). Other literature points out that the inequality between life expectancies based on income quartile is increasing over time (the years 2000-2010) [54]. This observation, coupled with the higher rate of transitioning for those below 1× poverty might point to focusing on those below the 2× poverty threshold to decrease income-related mortality or health discrepancies.

For all three lower income states, the largest discrepancy in variance of average remaining life expectancy occurs from the mid-30’s to late 50s. Individuals below the income thresholds have the highest variance, meaning they have a greater range of possibilities in life trajectory (5). Those above threshold have less variance, meaning individuals will more consistently reach their higher average remaining life expectancy.

Our results point to a phenomenon at mid-ages, that an individual in the higher (above 2× or 3× poverty) income state is less likely to enter the lower income state. And the higher the probability of stasis in the higher income state, the fewer individuals in the lower income states. However, there is higher inequality in life-expectancy, and variance at mid-ages (since at mid-ages the higher income state is less transient).

Our simulation findings are in agreement with “The most consistent finding in the literature… that the probability of entering poverty is much higher in young adulthood than in other stages of life” [6]. Rank (2015) (with PSID data) finds the occurrence of poverty is fairly widespread, between the ages of 25 and 60 they find 61.8 percent of the population will experience at least one year of relative poverty [55]. They found that “a predominate pattern is that individuals are often likely to experience one or two years of poverty, and then rise out of poverty, with perhaps an additional spell down the road.” When they looked at age groups they also found that those between the ages of 45 and 54 experienced the least incidence of poverty as opposed to the 25-34, 35-44 and 55-64 groups.

The dynamics of the poverty state is important; the less transient the higher income state, the larger the discrepancy in age-specific average remaining life expectancy and the variance. At young ages, where there is the highest probability of exiting the lower income state, there is a smaller difference in the average remaining life expectancy. Although more people are in poverty at young ages, they have more possibilities across their lifespan to change income state, thus there is less discrepancy in remaining life expectancy between income states.

We agree with Gillespie [56], that focusing on mid-ages to decrease income-related health disparities could help decrease lifespan inequality. Sandoval [57] combined period effects with looking at age classes to find fewer individuals enter poverty in their 40s and 50s as compared to other age classes, and found that the risk of poverty has been increasing over time (from 1970, 1980 to 1990), even for the low risk age-classes.

Further expansions of our model include examining how much of the changing poverty relationships with age are due to selection out of the cohort due to death. Additionally, comparative studies of age-specific poverty dynamics would yield interesting insight into different age-patterning across countries, regions and even data-sets [18, 51, 54]. Examining period effects in our simulations (for instance, [57] did between 1968 and 2000 and found that the life-course risk of poverty is increasing, especially in the 1990s) could allow the model to examine the effects of periods of economic turmoil. Adding an inter-generational element would be interesting as well, since there is an association between children’s and parent’s income [58]. This could be done for instance by using a Markov chain with rewards framework as Caswell (2015) has done [12]). And we could infinitely extend out matrix model dimensions to include additional states with hyperstate matrix models [24], for instance adding a state to reflect whether an individual has been in poverty in the past decade.

Our state-by-age-structured modeling of individuals undergoing stochastic entry and exit from 1×, 2× and 3× poverty yields a nuanced perspective of dynamic heterogeneity across the life course. We directly relate annual individual transience to state-specific disparities in life expectancy and variance. In doing so we extend the literature on individual poverty dynamics and stage-by-age matrix models. Our results suggest that dynamic heterogeneity in poverty and the transience of the poverty state is associated with income-related mortality disparities.

## Supporting information

S1 Theory (PDF)

S1 Appendix (PDF)

S2 Appendix (PDF)

S1 Tables (A) HRS logistic regression for each poverty threshold: Dependent Variable = Survival at time t+1. (B) HRS logistic regression: Dependent Variable = Poverty status at time t+1. (C) NLSY79-pre-1994 logistic regression for each poverty threshold: Dependent Variable = Survival at time t+1. (D) NLSY79-pre-1994 logistic regression for each poverty threshold: Dependent Variable = Poverty status at time t+1. (E) NLSY79-post-1994 logistic regression for each poverty threshold: Dependent Variable = Survival at time t+1. (F) NLSY79-post-1994 logistic regression for each poverty threshold: Dependent Variable = Poverty status at time t+1.
(PDF)

S1 Fig Pooled data from HRS and NLSY79. The dashed vertical line in each figure depicts where NLSY79 and HRS data are joined (at age 50). a, c, e: Weighted observations in each state at each age. b, d, f: Proportion of individuals in each state at each age. Each row examines the same population but differs in the threshold used to classify individuals into different states; a and b: below and above the standard poverty threshold; c and d: below and above 2× poverty threshold; e and f: below and above 3× poverty threshold which is close to national median income levels.
(TIF)

S2 Fig The distribution of non-response based on state and age. All panels are derived from HRS and NLSY79 data-sets. a, c, and e: The proportion of non response at age *x* + 1 based on being below an income threshold (red) or above an income threshold (blue) at age *x*. Income thresholds are defined as 1×, 2×, or 3× the ‘official’ poverty threshold, respectively. b, d, and e: Difference in non-response between the income categories (separated by 1×, 2×, or 3× poverty threshold, respectively) at each age.
(TIF)

S3 Fig Age distribution at each interview wave. For HRS (a) and NSLY79 (b).
(TIF)

S4 Fig Difference in months between interviews for HRS (left) and NSLY79 (right). HRS is normally distributed about 23.8 months. NLSY is binormally distrbuted about 12.4 and 24.1 months (pre and post-1994 interviews).
(TIF)
