# Supplementary material for: Poverty dynamics, poverty thresholds and mortality: An age-stage Markovian model
Source: PLoS One. 2018 May 16;13(5):e0195734. doi: 10.1371/journal.pone.0195734 (PMC5955488; doi:10.1371/journal.pone.0195734)
Supplement: S1 Theory — (PDF) [file pone.0195734.s001.pdf]

Supporting information

S1 Theory

Theory A: Projecting a cohort of newborns from birth onward

To see what happens to a cohort with a specific threshold (1×, 2× or 3× poverty) we must pass a cohort through the population projection matrix $\mathbf{L}$. For clarity we will refer to the two states as 'below or in poverty' and 'above poverty', although this technique is done three times for the 1×, 2× and 3× threshold. We start with a column vector for the population at time 0, $\mathbf{n(0)}$, where lets say $1,000,000$ individuals are born into the above poverty state (the first row represents the below poverty state).

$$\mathbf{n}(0) = \begin{bmatrix} 0 \\ 1,000,000 \\ 0 \\ \vdots \\ 0 \end{bmatrix} \tag{5}$$

At the first time step we pass our newborn cohort through the matrix by multiplying $\mathbf{L}n(0)$, and then we get a new population, $\mathbf{n}(1)$, which we then again pass through the matrix in a loop for all 100 time steps. In other words:

$$\mathbf{n}(x+1) = \mathbf{L}\mathbf{n}(x) \tag{6}$$

Which in a non-recursive form is synonymous to using $\mathbf{L}$ in equation [1]. The sum of the column vector $\mathbf{n}(x)$, $\sum_{i=1}^{202} n(x_i)$, represents the total number of individuals of the cohort who are alive at age x. In each $\mathbf{n}(x)$ there are only two nonzero elements: the first value (always on the odd row index) represents the population at age x that is above poverty, $a(x) = n(x_{2k-1})$. The second value (always on the even row index) represents the population at age x that is below poverty, $b(x) = n(x_{2k})$, here $k\epsilon[1, 2, 3..., 200, 101]$. Note that the vector $\mathbf{n}(x)$ has dimensions 202x1 since there are 101 time steps and 2 possible states. These values (of the population in either stage at each age) can be represented in vector form by a 2x1 vector denoted $\boldsymbol{\pi}(x)$ [1] which can be calculated with the following equation:

$$\boldsymbol{\pi}(x) = \frac{(\mathbf{1}_{101}^T \otimes \mathbf{I}_2)\mathbf{n}(x)}{||\mathbf{n}(x)||} \tag{7}$$

Where $\mathbf{1}_{101}$ is a column vector of ones of length 101, $\mathbf{I}_2$ is a square identity matrix of dimension 2 (since there are 2 stages, above and below a poverty threshold), and $\otimes$ represents the Kronecker product (meaning each element in $\mathbf{1}_{101}^T$ is multiplied by the matrix $\mathbf{I}_2$). Here $||\mathbf{n}(x)||$ is simply the sum of the vector $(\sum_{i=1}^{202} \mathbf{n}(x))$.

Survivorship to a particular age is defined as the total population at age x divided by the total initial cohort ($\mathbf{n(0)}$, here set at 1,000,000 individuals) [2].

$$\text{Survivorship to age } x = S(x) = \frac{\sum_{i=1}^{202} n(x_i)}{\sum_{i=1}^{202} n(0_i)} \tag{8}$$

The sum of the column vector $\mathbf{n(x)}$ can also be implemented mathematically by simply multiplying $\mathbf{n(x)}$ by a sum vector (a vector of 1s) of equal size. Hazard at a particular age is the instantaneous risk of mortality, the negative of the slope of the log(survivorship) (the rate of decrease in log(survivorship)), and can also be thought of as the instantaneous risk of dying. It can be calculated in a continuous time framework by [3]:

$$h = \log(-\log(S(x))) \tag{9}$$

Here we use the same equation even though we are working in a discrete time framework. These survivorship and hazard values are age specific rates for the entire cohort, comprised of both below income threshold and above income threshold individuals of age x. We are also interested in the proportion of individuals below the income threshold at each age, $(b(x)/(a(x) + b(x)))$, and the difference in average remaining life expectancy, for instance for above poverty and below poverty individuals at each age. In the next subsections we will show how the model can be used to look at remaining life expectancy for each income state, and the variability among individual trajectories of members of a cohort. All of these outputs are dependent on the input survival and transition parameter values, $s_1(x)$, $s_2(x)$, $t_{21}(x)$, $t_{22}(x)$.

Theory B: Markov chain analysis and the fundamental matrix: the average individual life expectancy

In the previous section the matrix $\mathbf{L}$ was used to project a cohort from age 0 to 100, this allows us to examine how the probabilities from the statistical models determine the demographic structure of the cohort. $\mathbf{L}$ can also be used in Markov chain analysis to calculate the fundamental matrix [4] and the average individual life expectancy in each state, which we will discuss here. Furthermore, stochastic individual life path trajectories where individuals enter and exit transient states and ultimately an absorbing state (death) can be calculated as well, and that will be discussed in the next section.

At age 0, remaining life expectancy is simply called life expectancy (the expected age at death for newborns), at every other age remaining life expectancy is the life expectancy conditional on having reached that age (the number of years an individual is expected to stay alive in the future, given that he/she survived to a particular age). For example, the remaining life expectancy at age 65 (which is conditional on survival to 65) is of special interest to the social security administration and to groups calculating health care spending for the elderly [5]. We can use our $\mathbf{L}$ matrix to calculate the fundamental matrix ($\mathbf{N}$) and then calculate the average individual remaining life expectancy values and related terms at each age [4, 6].

$$\text{The fundamental matrix} = \mathbf{N} = \mathbf{I} + \mathbf{L} + \mathbf{L}^2 + \ldots = \sum_{x=0}^{\infty} \mathbf{L}^x$$
$$= (\mathbf{I} - \mathbf{L})^{-1} \tag{10}$$
$$\text{Life expectancy} = \mathbf{j}'\mathbf{N} \tag{11}$$
$$\text{Variance of life expectancy} = \mathbf{j}'(2\mathbf{N}^2 - \mathbf{N}) - \mathbf{j}'(\mathbf{N}) \circ \mathbf{j}'(\mathbf{N}) \tag{12}$$
$$\text{Standard deviation of life expectancy} = (\text{Variance of life expectancy})^{0.5} \tag{13}$$

Where $\mathbf{I}$ is an identity matrix with the same dimensions as $\mathbf{L}$ and $\mathbf{j}$ is a column vector of 1s that gives the sum of each column when its transpose is multiplied by a matrix. ($\circ$ represent the Hadamard Product which is element-wise multiplication of $\mathbf{j}'\mathbf{N}$ and $\mathbf{j}'\mathbf{N}$). The fundamental matrix is essentially a series that converges to the inverse of the identity matrix minus $\mathbf{L}$ (eq.4). The fundamental matrix informs about the expected number of visits to a transient state. The sum of all of its columns yields the average remaining life expectancy conditional on survival for each age. Next we generate stochastic individual life path trajectories, as opposed to looking at the average trajectory values.

Theory C: Markov chain analysis: Generating stochastic individual life-paths, realizations

The matrix $\mathbf{L}$ with dimensions 202×202 describes part of an individual's movement through a Markov chain but an extra absorbing state, death, must be added to complete the description [6, 7]. Therefore, as in equation [2] one more column and row is added on to $\mathbf{L}$ which creates $\mathbf{P}$, which is column stochastic with dimensions 203×203. Here we show $\mathbf{P}$ with 2×2 unconditional state transition matrices $\mathbf{Q(x)}$ as above so note that for $\mathbf{L}$ each $0 = \begin{bmatrix} 0 & 0 \\ 0 & 0 \end{bmatrix}$ so the indexes of $\mathbf{Q}$ represent the number of state matrices at each age (age blocks) and each $\mathbf{m}(x)$ is a 1×2 row vector with $\mathbf{m}(101) = [1 \quad 1]$.

$$\mathbf{P} = \left(\begin{array}{cccccccc|c} 0 & 0 & 0 & \ldots & 0 & 0 & 0 & 0 & 0 \\ \mathbf{Q}(1) & 0 & 0 & \ldots & 0 & 0 & 0 & 0 & 0 \\ 0 & \mathbf{Q}(2) & 0 & \ldots & 0 & 0 & 0 & 0 & 0 \\ 0 & 0 & \mathbf{Q}(3) & \ldots & 0 & 0 & 0 & 0 & 0 \\ \vdots & \vdots & \vdots & \ddots & \vdots & \vdots & \vdots & \vdots & \vdots \\ 0 & 0 & 0 & \ldots & \mathbf{Q}(98) & 0 & 0 & 0 & 0 \\ 0 & 0 & 0 & \ldots & 0 & \mathbf{Q}(99) & 0 & 0 & 0 \\ 0 & 0 & 0 & \ldots & 0 & 0 & \mathbf{Q}(100) & 0 & 0 \\ \hline \mathbf{m}(1) & \mathbf{m}(2) & \mathbf{m}(3) & \ldots & \mathbf{m}(98) & \mathbf{m}(99) & \mathbf{m}(100) & \mathbf{m}(101) & 1 \end{array}\right) \tag{14}$$

Column 102 is the death absorbing state. Each element in $\mathbf{m}$, which is row 102, is the probability of death for that age and state:

$$\mathbf{m(x)} = \begin{bmatrix} 1 - s_1(x) & 1 - s_2(x) \end{bmatrix} \tag{15}$$

Where $1 - s_1(x)$ represents the below income threshold state, one-year probability of mortality at age x, and $1 - s_2(x)$ represents the annual probability of mortality for those in the higher income state at age x. Since $\mathbf{P}$ is an absorbing Markov chain it can be divided into two sets [6], a set of absorbing states, α, and a set of transient states, τ. The transient set is age-specific (for each x), since $\mathbf{P}$ is of state-by-age form:

$$\tau = \{2, 1\}_x, \qquad x \epsilon \{1, 2, ...101\} \tag{16}$$
$$\alpha = \{0\} \tag{17}$$

There is a pathway from each of the states in τ to α, since an individual can die at any state and age. The transient states are above poverty, '2' and below poverty, '1'. Age itself can also be viewed as a transient state and in that case there would be 202 transient states since for 101 different ages ($\{0, 1, 2...100\}$) an individual transitions (or is born) into either 2, 1, or the absorbing state 0. Let y be a column vector of $i\epsilon[1, 203]$ representing the probability distribution of states where $0 \leq y_i \leq 1$ and $\sum_i y_i = 1$. So the column vector $\mathbf{y}(0)$ represents an individual's probability at age 0 of being in state 0, 1, or 2. Then

$$y(x+1) = \mathbf{P}\mathbf{y}(x) \tag{18}$$

where

$$\mathbf{P}^x = \left(\begin{array}{c|c} \mathbf{L}^x & 0 \\ \hline m\sum_{n=0}^{x-1}\mathbf{L}^n & 1 \end{array}\right) \tag{19}$$

Since it is possible to reach the state 0, death, from every state; τ guarantees that the dominant eigenvalue of $\mathbf{L}$ is strictly less than one so $\lim_{x\to\infty}(\mathbf{L}^x) = 0$ [6]. This means that every individual will enter the absorbing state i.e. eventually die. The Markov chain approach gives rise to the generation of individual life paths based on the probabilities in the $\mathbf{L}$ matrix. The individual life paths are stochastic and we can observe different types of variability among individual trajectories. For instance, variability in age at death, age at first state transition, duration in a state, etc.
