# Supplementary material for: Poverty dynamics, poverty thresholds and mortality: An age-stage Markovian model
Source: PLoS One. 2018 May 16;13(5):e0195734. doi: 10.1371/journal.pone.0195734 (PMC5955488; doi:10.1371/journal.pone.0195734)
Supplement: S1 Appendix — (PDF) [file pone.0195734.s002.pdf]

S1 Appendix   One-period probability adjustments

One-period probability adjustments

The time between interviews for HRS is normally distributed about 23.8months (see S4 Fig). NLSY is bi-normally distributed about 12.4 and 24.1 months (S4 Fig), since after 1994 interviews were performed every 2 years as opposed to every one year. For HRS, NLSY-pre-1994, and NLSY-post-1994 we calculate regression parameters (see tables A-F in S1 Tables). The parameters are then logit transformed to calculate probability of survival between t and $t+i$, and probability of transitioning between t and $t+i$. We then adjust i to be 12 months, a one year period.

To calculate our one-year state transition rates from one age to the next age, $t(x)_{21}$ and $t(x)_{22}$, from the logit transformed regression coefficients we use the following equation:

$$\begin{bmatrix} t_{11} & t_{12} \\ t_{21} & t_{22} \end{bmatrix} = \begin{bmatrix} r_{11} & r_{12} \\ r_{21} & r_{22} \end{bmatrix}^{12/i} \tag{20}$$

Where r_{21} and r_{22} are the logit transformed regression parameters and $r_{11} = 1 - r_{21}$ and $r_{12} = 1 - r_{22}$. Note, every variable is a function of age, x, but we leave the x out here for clarity. i is either 12.4, 24.1, or 23.8 depending on the data-set, and for our matrix and power values we have a defined root. (In our case our matrices are nonsingular (with nonzero determinants, nonzero eigenvalues) and since all eigenvalues are positive and the matrix is real there is a distinguished root that is real. (Theorem 2.5 Higham and Al-Mohy (2010) [8])

To calculate our one-year survival rates for each age, $s(x)_1$ and $s(x)_2$, from the logit transformed regression coefficients, c_1 and c_2, we estimate with the following equations:

$$c_1 = s_1^2 t_{11} + s_1 s_2 t_{21} \tag{21}$$

$$c_2 = s_2^2 t_{22} + s_2 s_1 t_{12} \tag{22}$$

In this case, we estimate that i = 2 and use this formula for NLSY-post-1994 and HRS. We use the transition probabilities, t_{11} etc., calculated from above. Here c_1, the logit transformed regression coefficient, is the probability of survival between t and $t+i \approx t+2$, for those below threshold. And c_2 is the same for those above income threshold. Again, every variable is a function of age x. We estimate that for NLSY-pre-1994 $c_1 = s_1$.
