# Supplementary material for: Poverty dynamics, poverty thresholds and mortality: An age-stage Markovian model
Source: PLoS One. 2018 May 16;13(5):e0195734. doi: 10.1371/journal.pone.0195734 (PMC5955488; doi:10.1371/journal.pone.0195734)
Supplement: S2 Appendix — (PDF) [file pone.0195734.s003.pdf]

S2 Appendix Empirical data

Empirical data and non-response

After pooling and then combining data from NLSY79 and HRS (S1 Fig) we observe an increasing proportion of individuals in poverty as the poverty threshold is raised.

Since the pooled logistic regression approach omits missing data we addressed the issue of whether non-response was itself associated with poverty status [9]. In S2 Fig we show the proportion of missing observations at age $x + i$ based on state at age x, and the difference in non-response between each state. There is slightly lower response by those in the lower income states for all three thresholds. The (weighted) difference does not seem to be dependent on age so we assume that for our analysis here the impact on the results are minimal [10].

References

1. Caswell H, de Vries C, Hartemink N, Roth G, van Daalen S. Age$\times$stage-classified demographic analysis: a comprehensive approach. In review. 2017;.

2. Fox GA. Failure Time Analysis: Studying Time to Events and Rates at Which Events Occur. New York: Oxford University Press, Inc; 2001.

3. Venables WN, Ripley BD. Modern Applied Statistics with S. 4th ed. New York: Springer-Verlag; 2002.

4. Kemeny JG, Snell JL. Finite Markov Chains. Springer-Verlag; 1976.

5. Lubitz J, Cai L, Kramarow E, Lentzner H. Health, Life Expectancy, and Health Care Spending Among the Elderly. The New England Journal of Medicine. 2003;349(11):1048–55. doi:10.1056/NEJMsa020614.

6. Caswell H. Matrix Population Models. 2nd ed. Sunderland, Massachusetts: Sinauer Associates, Inc; 2001.

7. Caswell H. Matrix Models and Sensitivity Analysis of Populations Classified by Age and Stage: A Vec-Permutation Matrix Approach. Theoretical Ecology. 2012;5:403–417. doi:10.1007/s12080-011-0132-2.

8. Higham NJ, Al-mohy AH. Computing Matrix Functions. Acta Numerica(10); 2010.

9. Carlin JB, Wolfe R, Coffey C, Patton GC. Analysis of Binary Outcomes in Longitudinal Studies using Weighted Estimating Equations and Discrete-time Survival Methods; Prevalence and Incidence of Smoking in an Adolescent Cohort. Statistics in Medicine. 1999;18(December 1998):2655–2679.

10. Horton NJ, Kleinman KP. Much Ado About Nothing. The American Statistician. 2007;61(1):79–90. doi:10.1198/000313007X172556.
