# Supplementary material for: Poverty dynamics, poverty thresholds and mortality: An age-stage Markovian model
Source: PLoS One. 2018 May 16;13(5):e0195734. doi: 10.1371/journal.pone.0195734 (PMC5955488; doi:10.1371/journal.pone.0195734)
Supplement: S1 Tables — (A) HRS logistic regression for each poverty threshold: Dependent Variable = Survival at time t+1. (B) HRS logistic regression: Dependent Variable = Poverty status at time t+1. (C) NLSY79-pre-1994 logistic regression for each poverty threshold: Dependent Variable = Survival at time t+1. (D) NLSY79-pre-1994 logistic regression for each poverty threshold: Dependent Variable = Poverty status at time t+1. (E) NLSY79-post-1994 logistic regression for each poverty threshold: Dependent Variable = Survival at time t+1. (F) NLSY79-post-1994 logistic regression for each poverty threshold: Dependent Variable = Poverty status at time t+1. (PDF) [file pone.0195734.s004.pdf]

S1 Tables Regression coefficients

Table S1.A. HRS logistic regression for each poverty threshold: Dependent Variable = Survival at time t+1

**Predictor**	**Estimate**	Std. Error	t value	$Pr(>\|t\|)$
Intercept (Below 1× poverty)	7.311	0.353	20.717	<2e-16 ***
Slope (Below 1× poverty)	-0.068	0.004	-14.727	<2e-16 ***
Δ Intercept (Above 1× poverty)	2.470	0.389	6.349	2.17e-10***
Δ Slope (Above 1× poverty)	-0.026	0.005	5.229	1.71e-07***
Intercept (Below 2× poverty)	7.460	0.233	32.017	<2e-16 ***
Slope (Below 2× poverty)	-0.069	0.003	-22.920	<2e-16 ***
Δ Intercept (Above 2× poverty)	2.804	0.306	9.166	<2e-16 ***
Δ Slope (Above 2× poverty)	-0.031	0.004	-7.986	1.41e-15***
Intercept (Below 3× poverty)	7.920	0.200	39.621	<2e-16 ***
Slope (Below 3× poverty)	-0.074	0.002	-28.719	<2e-16 ***
Δ Intercept (Above 3× poverty)	2.695	0.310	8.694	<2e-16***
Δ Slope (Above 3× poverty)	-0.030	0.004	-7.454	9.16e-14***

$^{***}p < 0.01$, $^{**}p < 0.05$, $^{*}p < 0.1$

Table S1.B. HRS logistic regression: Dependent Variable = Poverty status at time t+1

**Predictor**	**Estimate**	Std. Error	t value	$Pr(>\|t\|)$
Intercept (Below 1× poverty)	0.354	0.187	1.893	0.058*
Slope (Below 1× poverty)	-0.007	0.003	-2.879	0.004***
Δ Intercept (Above 1× poverty)	2.418	0.235	10.307	<2e-16***
Δ Slope (Above 1× poverty)	0.009	0.003	2.774	0.005***
Intercept (Below 2× poverty)	-0.108	0.125	-0.861	0.389
Slope (Below 2× poverty)	-0.012	0.002	-6.784	1.17e-11***
Δ Intercept (Above 2× poverty)	3.935	0.161	24.483	<2e-16***
Δ Slope (Above 2× poverty)	-0.017	0.002	-7.567	3.86e-14***
Intercept (Below 3× poverty)	-0.211	0.110	-1.913	0.056*
Slope (Below 3× poverty)	-0.016	0.002	10.194	<2e-16***
Δ Intercept (Above 3× poverty)	4.257	0.147	29.003	<2e-16***
Δ Slope (Above 3× poverty)	-0.025	0.002	-11.987	<2e-16***

$^{***}p < 0.01$, $^{**}p < 0.05$, $^{*}p < 0.1$

Table S1.C. NLSY79-pre-1994 logistic regression for each poverty threshold: Dependent Variable = Survival at time t+1

**Predictor**	**Estimate**	Std. Error	t value	$Pr(>\|t\|)$
Intercept (Below 1× poverty)	7.434	1.400	5.307	1.12e-07 ***
Slope (Below 1× poverty)	-0.046	0.051	-0.890	0.373
Δ Intercept (Above 1× poverty)	0.107	1.840	0.058	0.954
Δ Slope (Above 1× poverty)	0.022	0.067	0.320	0.749
Intercept (Below 2× poverty)	7.084	1.181	5.997	2.01e-09 ***
Slope (Below 2× poverty)	-0.028	0.044	-0.661	0.508
Δ Intercept (Above 2× poverty)	1.015	1.937	0.524	0.600
Δ Slope (Above 2× poverty)	-0.009	0.070	-0.127	0.899
Intercept (Below 3× poverty)	8.389	1.120	7.484	7.25e-14 ***
Slope (Below 3× poverty)	-0.067	0.040	-1.660	0.096*
Δ Intercept (Above 3× poverty)	-2.284	2.179	-1.048	0.294
Δ Slope (Above 3× poverty)	0.103	0.080	1.290	0.197

$^{***}p < 0.01$, $^{**}p < 0.05$, $^{*}p < 0.1$

Table S1.D. NLSY79-pre-1994 logistic regression for each poverty threshold: Dependent Variable = Poverty status at time t+1

**Predictor**	**Estimate**	Std. Error	t value	$Pr(>\|t\|)$
Intercept (Below 1× poverty)	1.220	0.188	6.462	1.04e-10 ***
Slope (Below 1× poverty)	-0.056	0.007	-8.027	1.01e-15 ***
Δ Intercept (Above 1× poverty)	-0.032	0.255	-0.128	0.898
Δ Slope (Above 1× poverty)	0.124	0.009	12.982	< 2e-16 ***
Intercept (Below 2× poverty)	0.664	0.137	4.850	1.24e-06***
Slope (Below 2× poverty)	-0.059	0.005	-11.515	< 2e-16***
Δ Intercept (Above 2× poverty)	-0.606	0.199	-3.043	0.00234***
Δ Slope (Above 2× poverty)	0.139	0.007	18.640	<2e-16***
Intercept (Below 3× poverty)	-0.107	0.122	-0.881	0.378
Slope (Below 3× poverty)	-0.049	0.004	-10.720	<2e-16***
Δ Intercept (Above 3× poverty)	-0.597	0.188	-3.180	0.001***
Δ Slope (Above 3× poverty)	0.135	0.006	19.457	<2e-16***

$^{***}p < 0.01$, $^{**}p < 0.05$, $^{*}p < 0.1$

Table S1.E. NLSY79-post-1994 logistic regression for each poverty threshold: Dependent Variable = Survival at time t+1

**Predictor**	**Estimate**	Std. Error	t value	$Pr(>\|t\|)$
Intercept (Below 1× poverty)	8.527	0.999	8.539	<2e-16 ***
Slope (Below 1× poverty)	-0.105	0.023	-4.490	7.14e-06 ***
Δ Intercept (Above 1× poverty)	0.203	1.261	0.161	0.872
Δ Slope (Above 1× poverty)	0.028	0.029	0.941	0.347
Intercept (Below 2× poverty)	8.986	0.749	11.995	<2e-16 ***
Slope (Below 2× poverty)	-0.108	0.017	-6.240	4.41e-10 ***
Δ Intercept (Above 2× poverty)	-0.389	1.232	-0.316	0.752
Δ Slope (Above 2× poverty)	0.039	0.028	1.371	0.170
Intercept (Below 3× poverty)	9.265	0.717	12.911	<2e-16 ***
Slope (Below 3× poverty)	-0.107	0.016	-6.414	1.43e-10***
Δ Intercept (Above 3× poverty)	-0.779	1.332	-0.585	0.559
Δ Slope (Above 3× poverty)	0.041	0.031	1.315	0.188

$^{***}p < 0.01$, $^{**}p < 0.05$, $^{*}p < 0.1$

Table S1.F. NLSY79-post-1994 logistic regression for each poverty threshold: Dependent Variable = Poverty status at time t+1

**Predictor**	**Estimate**	Std. Error	t value	$Pr(>\|t\|)$
Intercept (Below 1× poverty)	0.650	0.236	2.751	0.00595 ***
Slope (Below 1× poverty)	-0.024	0.005	-4.244	2.20e-05 ***
Δ Intercept (Above 1× poverty)	2.153	0.306	7.029	2.11e-12 ***
Δ Slope (Above 1× poverty)	0.030	0.007	4.037	5.42e-05 ***
Intercept (Below 2× poverty)	-0.329	0.173	-1.903	0.057*
Slope (Below 2× poverty)	-0.013	0.004	-3.229	0.001***
Δ Intercept (Above 2× poverty)	2.451	0.245	9.998	<2e-16***
Δ Slope (Above 2× poverty)	0.022	0.006	3.701	0.000215***
Intercept (Below 3× poverty)	-0.876	0.147	-5.935	2.95e-09***
Slope (Below 3× poverty)	-0.009	0.003	-2.588	0.009***
Δ Intercept (Above 3× poverty)	1.935	0.221	8.751	<2e-16***
Δ Slope (Above 3× poverty)	0.032	0.005	5.889	3.91e-09***

$^{***}p < 0.01$, $^{**}p < 0.05$, $^{*}p < 0.1$
