# Supplementary figures and images for: Poverty dynamics, poverty thresholds and mortality: An age-stage Markovian model
Source: PLoS One. 2018 May 16;13(5):e0195734. doi: 10.1371/journal.pone.0195734 (PMC5955488; doi:10.1371/journal.pone.0195734)

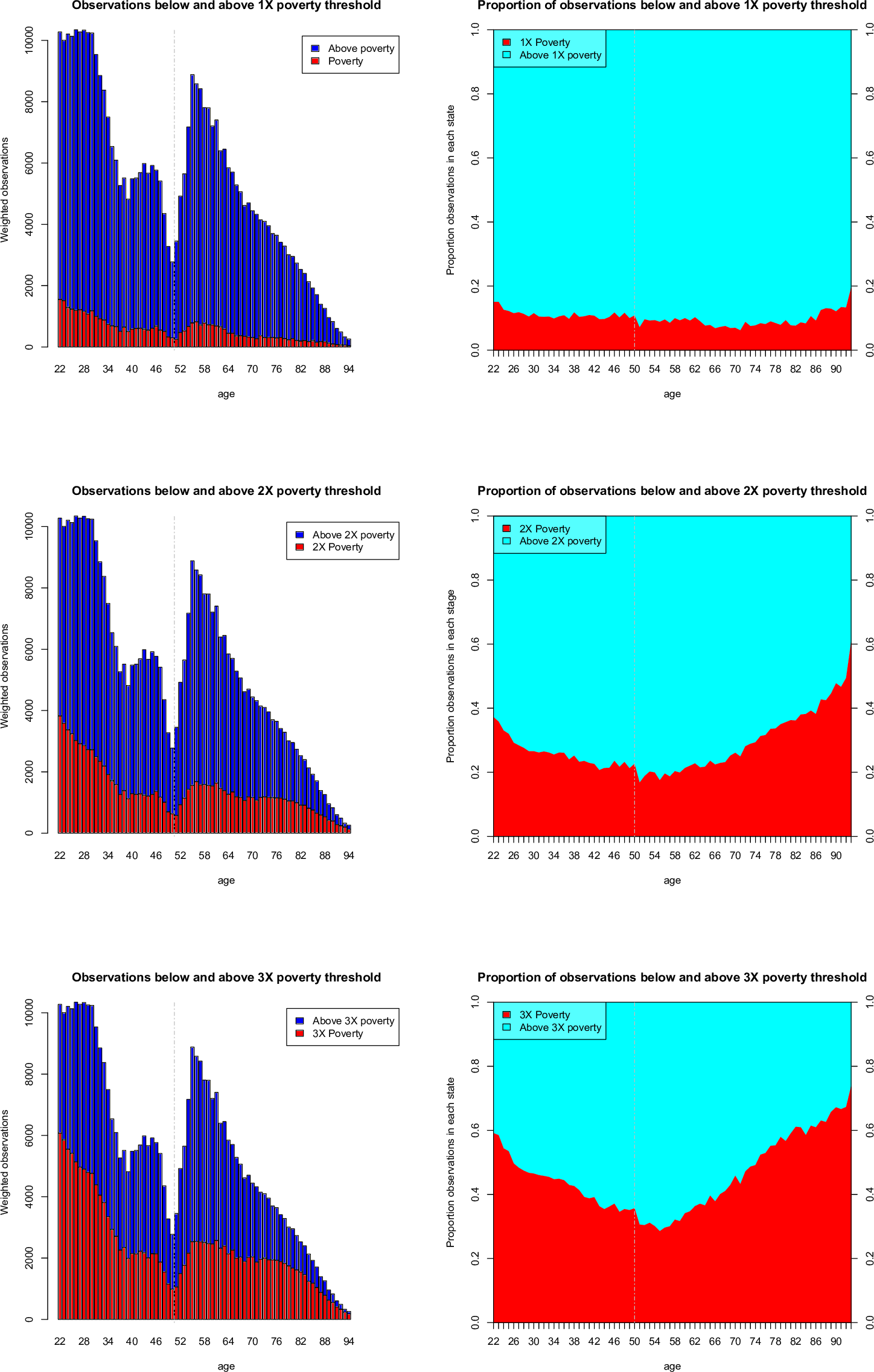

Supplement: S1 Fig — The dashed vertical line in each figure depicts where NLSY79 and HRS data are joined (at age 50). a, c, e: Weighted observations in each state at each age. b, d, f: Proportion of individuals in each state at each age. Each row examines the same population but differs in the threshold used to classify individuals into different states; a and b: below and above the standard poverty threshold; c and d: below and above 2× poverty threshold; e and f: below and above 3× poverty threshold which is close to national median income levels. (TIF) [file pone.0195734.s005.tif]

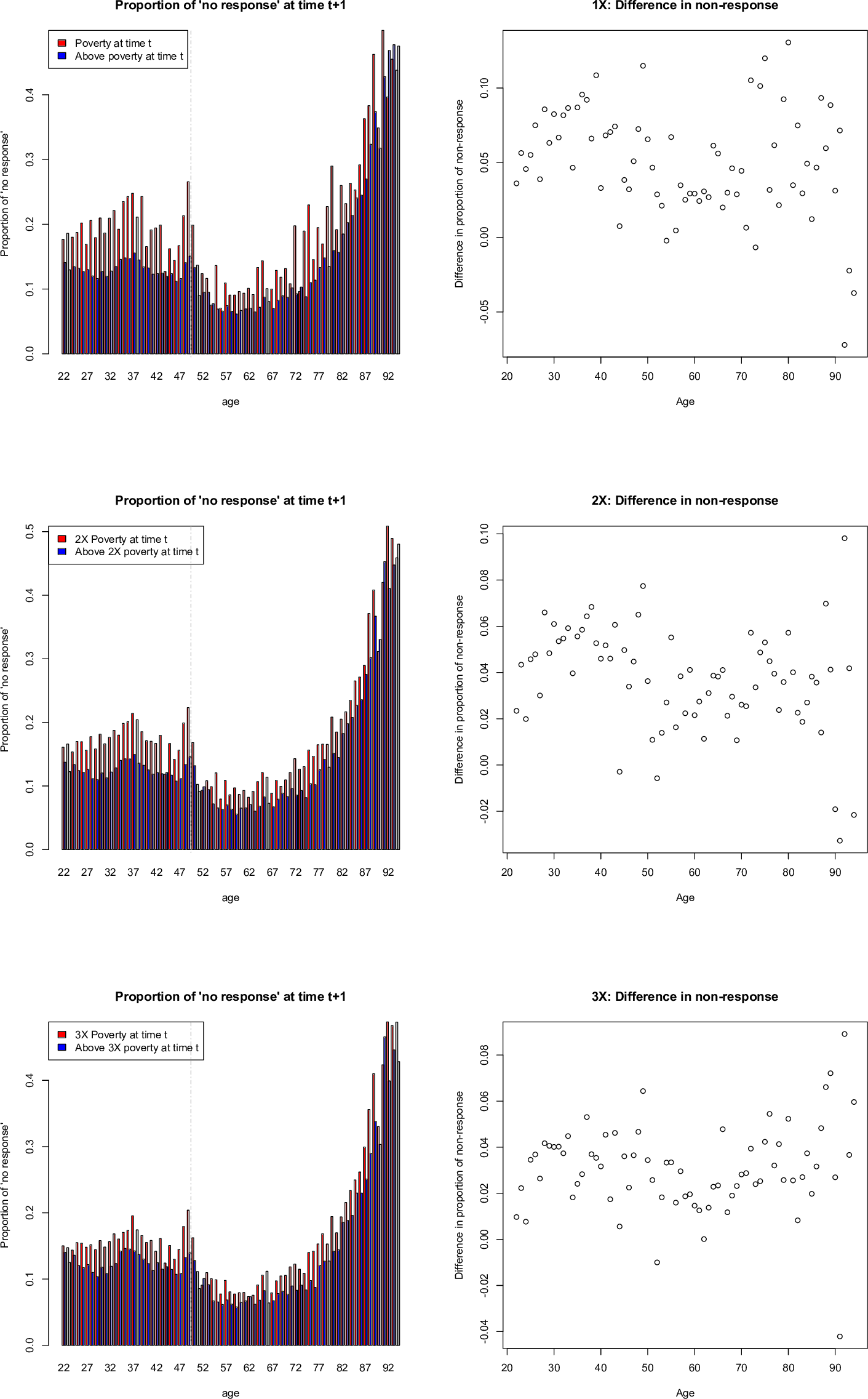

Supplement: S2 Fig — All panels are derived from HRS and NLSY79 data-sets. a, c, and e: The proportion of non response at age x + 1 based on being below an income threshold (red) or above an income threshold (blue) at age x. Income thresholds are defined as 1×, 2×, or 3× the ‘official’ poverty threshold, respectively. b, d, and e: Difference in non-response between the income categories (separated by 1×, 2×, or 3× poverty threshold, respectively) at each age. (TIF) [file pone.0195734.s006.tif]

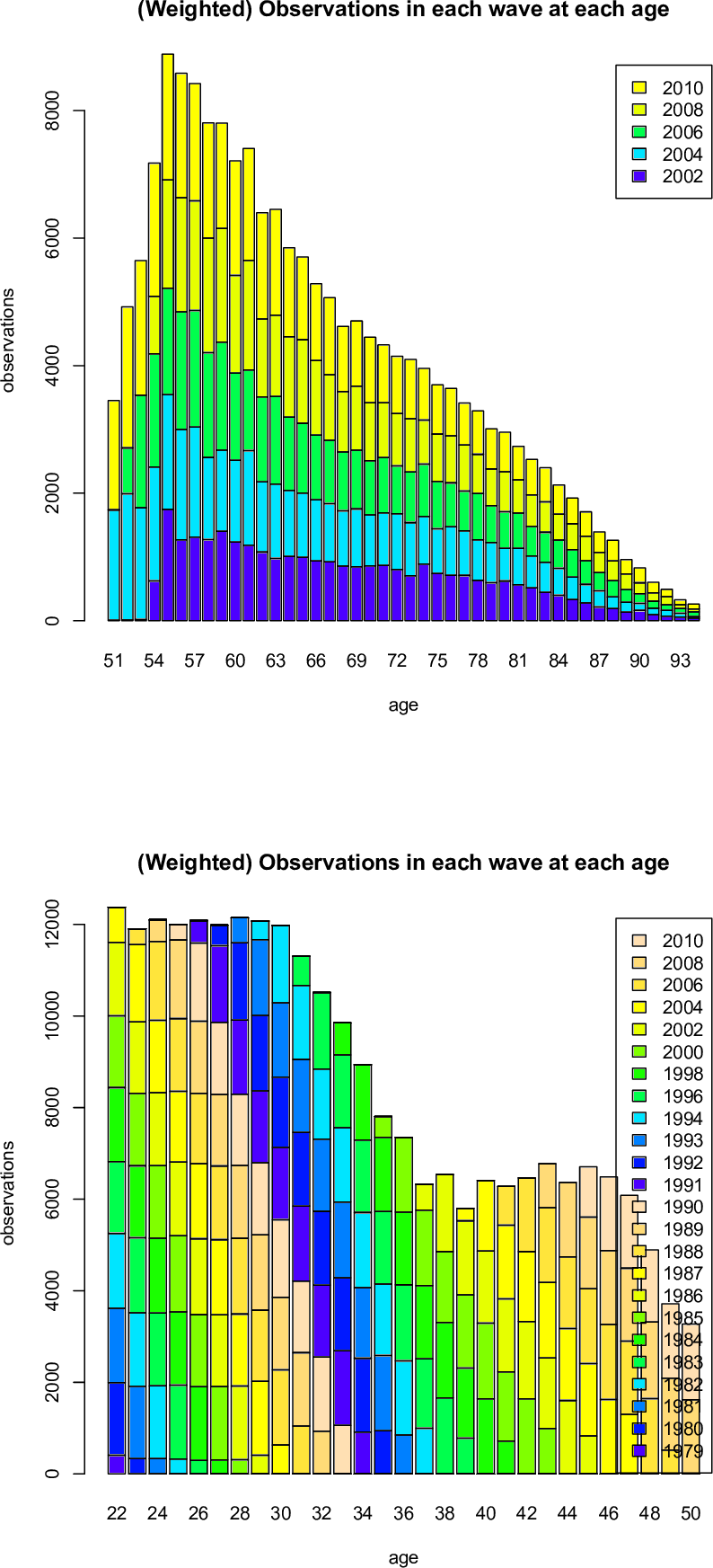

Supplement: S3 Fig — For HRS (a) and NSLY79 (b). (TIF) [file pone.0195734.s007.tif]

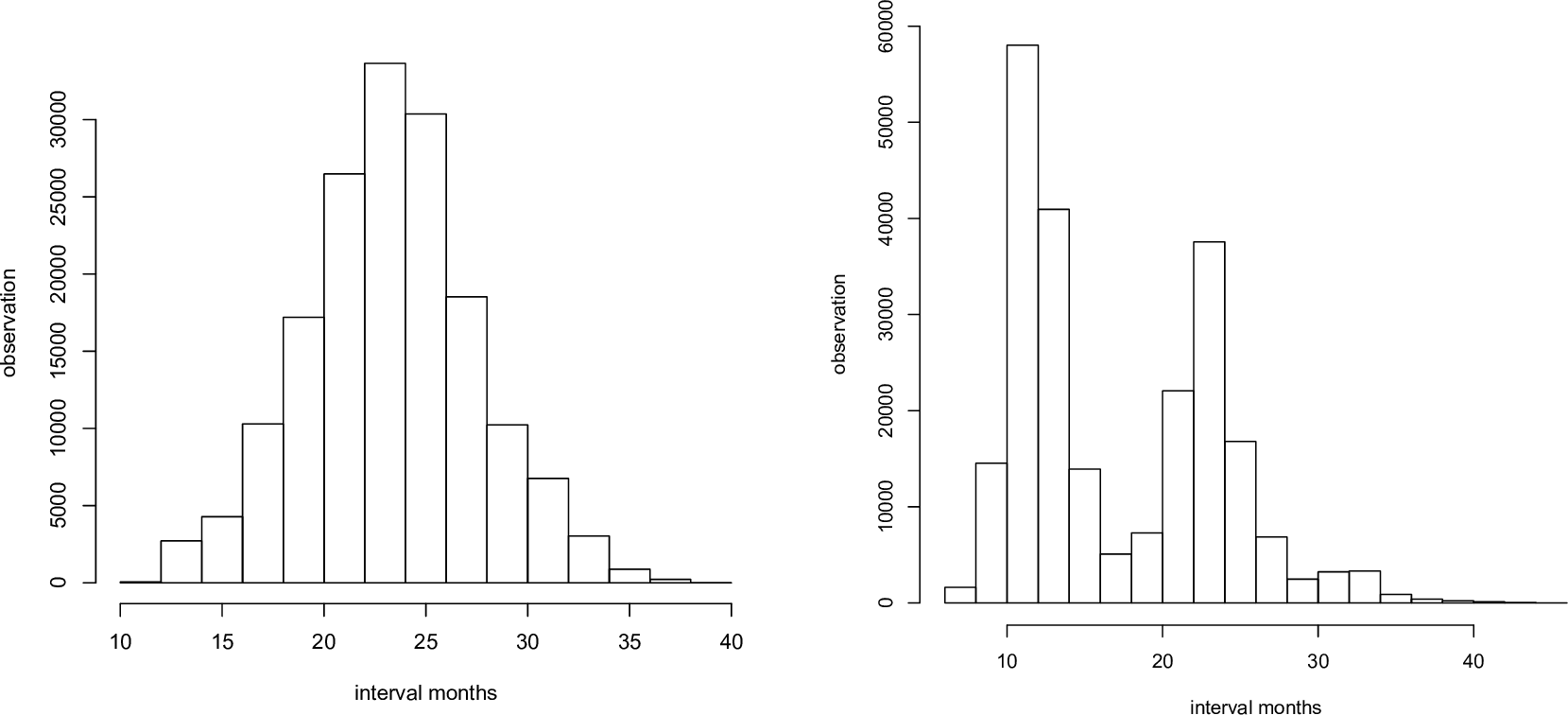

Supplement: S4 Fig — HRS is normally distributed about 23.8 months. NLSY is binormally distrbuted about 12.4 and 24.1 months (pre and post-1994 interviews). (TIF) [file pone.0195734.s008.tif]
